# A New Method of Predicting the Structural and Mechanical Change of Materials during Extrusion by the Method of Multiple Plastic Deformations

**DOI:** 10.3390/ma14102594

**Published:** 2021-05-16

**Authors:** Marta Harničárová, Jan Valíček, Milena Kušnerová, Zuzana Palková, Ivan Kopal, Cristina Borzan, Milan Kadnár, Stanislav Paulovič

**Affiliations:** 1 Department of Electrical Engineering, Automation and Informatics, Faculty of Engineering, Slovak University of Agriculture in Nitra, Tr. A. Hlinku 2, 949 76 Nitra, Slovakia; jan.valicek@uniag.sk (J.V.); zuzana.palkova@uniag.sk (Z.P.); stanislav.paulovic@uniag.sk (S.P.); 2 Department of Mechanical Engineering, Faculty of Technology, Institute of Technology and Business in České Budějovice, Okružní 10, 370 01 České Budějovice, Czech Republic; kusnerova.milena@mail.vstecb.cz; 3 Department of Numerical Methods and Computational Modeling, Faculty of Industrial Technologies in Púchov, Alexander Dubček University of Trenčín, Ivana Krasku 491/30, 020 01 Púchov, Slovakia; ivan.kopal@tnuni.sk; 4 Department of Manufacturing Engineering, Machine Building Faculty, Technical University of Cluj-Napoca, B-dul Muncii no. 103–105, 400641 Cluj-Napoca, Romania; cristina.borzan@tcm.utcluj.ro; 5 Department of Machine Design, Faculty of Engineering, Slovak University of Agriculture in Nitra, Tr. A. Hlinku 2, 949 76 Nitra, Slovakia; milan.kadnar@uniag.sk

**Keywords:** structural and mechanical changes, ECAP, extrusion, grain size, copper

## Abstract

The formulation of the Hall–Petch relationship in the early 1950s has raised immense interest in studying the influence of the grain size of solid materials on their properties. Grain refinement can be achieved through extreme deformation. In the presented study, Equal-Channel Angular Pressing (ECAP) was successfully applied to produce an ultrafine-grained microstructure in a pure commercial Cu of 99.9 wt%. Samples were processed by ECAP at 21 °C for six passes via route A. A new equation of equilibrium that allows the exact determination of the number of extrusions and other technological parameters required to achieve the desired final grain size has been developed. The presented research also deals, in a relatively detailed and comparative way, with the use of ultrasound. In this context, a very close correlation between the process functions of extrusion and the speed of longitudinal ultrasonic waves was confirmed.

## 1. Introduction

The microstructure is the structure of a material and is one of the most important factors that affect the physical and mechanical properties, in some cases, even more than chemistry. By knowing the microstructure, we can predict and control the properties of engineering materials. Microstructure evolves during the thermo-mechanical processing of materials [1]. The basic properties of the material (melting point, Young’s modulus, density, hardness, plasticity, fracture toughness, conductivity) are key information for scientists to select the right way of processing the materials to reach the desired structure. The study of the microstructure is, therefore, the subject of intensive research. A short time ago, the science of processing materials had acquired new importance. Grain refinement is the right approach to increase the yield strength or to improve fatigue life. Grain refinement is the process of reduction in the size of grains. The size of grains affects the strength of any material according to the Hall–Petch equation [2]. The smaller the grain size, the higher the strength becomes. Grain refinement is caused by severe plastic deformation (SPD), various processes have been developed which can induce SPD, these include equal channel angular pressing [3], equal channel multi-angular pressing [4], multiple forging [5], high-pressure torsion straining [6] or novel method differential velocity sideways extrusion [7], etc. The total number of currently known SPD techniques exceeds 60 and the development of new methods may be expected in the future [3].

For the development of the above and newly developed methods, understanding the mechanisms of grain refinement by plastic deformation is essential, especially in the field of nanometres. Many published works have shown that the process of grain refinement during plastic deformation has its origin in dislocation activities. This has been demonstrated in metals and their alloys, especially with the FCC lattice (Al, Cu, Ni, etc.), but also with the BCC lattice (Fe, low carbon steels) and the HCP lattice (Ti). It is generally accepted that plastic deformation induces a generation of high-density lattice dislocations in the original grains. Grain refinement in metals by plastic deformation generally depends on several factors. In addition to the already mentioned structure of the crystal lattice, several factors must be mentioned: state of the structure before deformation (initial grain size, character of microstructure), presence of second phase particles, deformation temperature and strain rate, deformation conditions (strain size and strain path). Methods of creating extreme plastic deformation (SPD) belong to the so-called “top-down” methods of preparation of ultrafine-grained (UFG) and nanocrystalline (NC) solid materials. Materials prepared by SPD usually have grains in the size range of 100–1000 nm. Materials after SPD in many cases exhibit excellent mechanical properties, such as high superplasticity at high strain rates and medium or elevated temperatures, high strength and relatively good ductility [8].

### 1.1. Properties of Materials Prepared by SPD

The application of SPD significantly changes the mechanical properties of metals and their alloys, especially their strength and ductility. Changes in mechanical properties (strength, hardness, ductility, fracture toughness, fatigue, creep, superplastic behavior), changes in internal damping, corrosion properties, magnetic and other properties were observed. A large number of papers have been published describing the results obtained in forming mainly by ECAP and HPT (high-pressure torsion) methods of aluminum and its alloys, nickel, copper and later pure iron, steel, titanium and other metals and alloys.

It is well known that greater plastic deformation induced by conventional methods of forming metals and their alloys, such as drawing, rolling or extrusion, results in a significant reduction in grain size in the structure and a consequent increase in strength and hardness. However, this increase is usually associated with a decrease in ductility due to the fact that the microstructure consists mainly of grains that have low-angle boundaries phenomenon. This phenomenon has been observed in many cases even with the application of SPD. Three factors limit ductility in ultra-fine and nanostructured materials. These include for example artifacts created during material preparation, instability under tensile loading, nucleation of cracks or instability in their propagation.

Elongation, which is the ability of a material to change shape without breaking, in a homogeneous material depends on the amount of strain hardening and sensitivity to strain rate. High values of these parameters help to suppress the onset of localized deformation (throttling) under tensile stress and thus increase ductility. Deformation strengthening is caused by the accumulation of crystal lattice defects such as dislocations, which makes further deformation more difficult. However, in nanostructured metals, the accumulation of dislocations is difficult because the grains are too small. Zero strain hardening has been observed for many nanostructured metals [8].

### 1.2. Equal Channel Angular Pressing

One of the most popular techniques is the ECAP (Equal Channel Angular Pressing) method. A founder of this method is considered Segal et al. [3,9]. The most famous name associated with the ECAP technology is Valiev et al. [10,11,12], who dealt with pure metals and alloys. Forming the ultrafine grains or even nanostructure needs is done through the specially designed equipment, where the plastic deformation occurs at higher strains. A metal billet is pressed through a die containing two channels with the same cross-sections and intersecting at an angle *Φ* in the range of 90–120°. When the billet is pressed, it undergoes severe shear deformation. Refinement is achieved by repeating the pressings for several passes [13,14]. There are four basic different processing routes. The difference is given by way of the billet rotation and the subsequent pass. There are many works performing a complex study of ECAP that should help to understand the process in several aspects. ECAP technology remains the most popular method used to create an ultrafine-grained structure. It has considerable potential for commercialization because of the expected enlargement of shaped billets and the development of continuous forming technologies. Therefore, further research in the field of die optimization and finding optimal regimes and deformation paths is required. The “classic” implementation of the ECAP method has inspired many researchers to create several modifications. Segal patented movable channel walls to reduce friction [15], Stecher and Thomson patented a die complemented by a rotating cylinder to reduce friction [16], Markusev et al. [17] patented a die with channels of different cross-sections in which the billet changes its cross-section in the outlet channel. In this case, the used pressure increases, and the life of the die decreases. To increase the deformation of the billet during one pass through the die, Liu et al. [18] designed the S-shaped channel. This arrangement is equivalent to path C, which assumes rotation of the billet about 180° between each pass in a conventional two-channel die. Krallics et al. [19] designed the U-shaped channel equivalent to path A (the billet does not rotate). Using the U-shaped channel doubles deformation and increases ECAP productivity. Raab et al. [20] used backpressure in the ECAP method in the outlet channel. The backpressure was created by placing a viscoplastic material (lead) in an outlet channel provided with a throttle insert. The use of backpressure had a positive effect on the strength and ductility of wrought copper. The design and construction of equipment for ECAP were studied through a series of finite element analyses [21,22,23]. Parshikov et al. [24] analyzed the most common technical problems that this technology must face. In order to reduce irregular shear strain distribution, which ECAP typically obtains, attention must be paid to die geometry and friction conditions. Similar research was also done by Prangnell et al. [25] To study the structures of the materials created by ECAP is fundamental because such a structure has unique properties. Valiev et al. subjected commercial pure copper to 16 passes of ECAP by route Bc. Coarse-grained Cu has a low yield strength but shows significant strain hardening and high fracture toughness. After 16 passes, it has been observed that there was both an increase in strength and ductility whereas the increase in ductility was more significant than the increase in strength. Such results have been achieved for the first time. Similar results were obtained on Ti.

Tong et al. [26] present their experience with ECAP pressing of the Mg–Zn–Ca alloy and analyses the influence of ECAP routes on the Mg–Zn–Ca alloy microstructure and its mechanical properties. There have been many other studies investigating the influence of ECAP routes on the microstructure and mechanical properties of different materials; 3003 Al alloy was studied by Lee et al. [27], pure Ti by Stolyarov et al. [28] or AZ80 Mg alloy by Gajanan et al. [29]. Nanostructured pure copper seems to be a very interesting material in many areas, mainly in electronics. Experimental research described in the paper by Irfan et al. [30] was carried out to investigate the influence of ECAP on erosion-corrosion of pure copper. The most important parameter affecting the behavior of copper after ECAP was the impacting angle and velocity. A combination of high strength and high ductility of nanostructured pure Cu after ECAP was found in [31]. The issue of deformation homogeneity in nanostructured pure Cu produced by ECAP was studied by Bhargava et al. [32] and Švec et al. [33]. They found out that a few passes and friction conditions influence, to a large extent, the strain inhomogeneity. Results on mechanical properties and microstructure of pure copper subjected to ECAP were also introduced by Zhang et al. [34]. After three passes, their results exhibit an increase in tensile strength, and a significant increase of hardness was reached after the three passes, then it started to decrease. 

### 1.3. Motivation of the Study

The objective of our experiment was to propose a new methodology, which would improve the efficiency of SPD processes through a new equation of equilibrium achieved in the elbow channel. Up to now, there are not enough analytical equations available to determine the state of equilibrium. Therefore, elastic-plastic theory must be utilized in the assessment of extrusion processes. The structure of the equations governing elastic-plastic deformation requires a quite different analytical solution procedure. Obtaining an equilibrium equation for ECAP technology is important for the subsequent identification and prediction of forces and stresses, which are needed to obtain refinement of the structure of the investigated material. It considers the causes, consequences and the balance in the extrusion process when creating new materials to obtain them on a nano basis. The next step in the research of ultrafine-grained materials seems to be the continuation of the development of ECAP devices with higher productivity. In parallel with the development of the devices, it is necessary to conduct optimal research on the technology used for formation by different methods for selected types of materials to achieve required mechanical and especially strength properties.

## 2. Materials and Methods

### 2.1. Experimental Procedures 

Changes in the basic input parameters chosen in this way were measured according to the valid Czech national standards in the cooperating accredited laboratory at the VÚHŽ Research Institute (Research Institute of Iron Metallurgy, Dobrá, Czech Republic). The actual extrusion on the ECAP device was performed using the equipment in the laboratory at VŠB-Technical University in Ostrava, Czech Republic.

**Figure 1:**
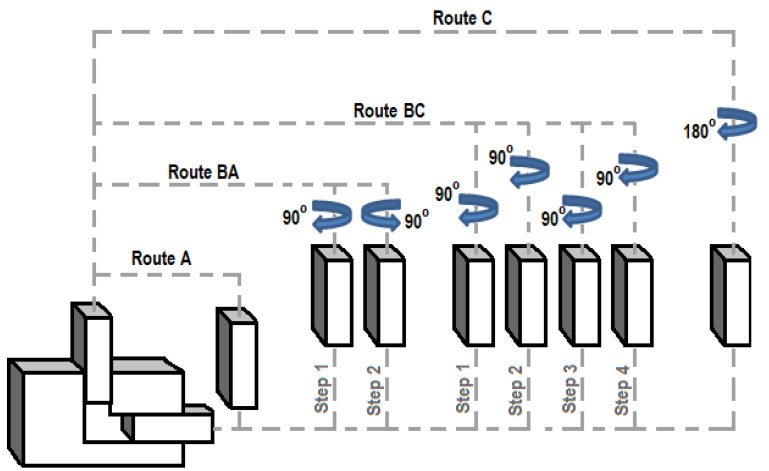
Routes creating various changes in the material during ECAP extrusion.

The principle of the Equal Channel Angular Pressing (ECAP) is shown in Figure 1, where the single routes are described as follows:

1. Route A: the sample is repeatedly compressed without rotation;
2. Route BA: the sample is rotated 90° in an alternative direction between each pass;
3. Route BC: the sample rotates 90° in the same direction;
4. Route C: the sample rotates 180° between each pass.

The choice of the deformation path and the number of passes are critical factors for the development of the microstructure and its resulting properties. When extruding a sample through successive passes, it is possible to change the shear stress characteristics by rotating it after each pass.

Commercially pure copper Cu 99.9% was chosen as the test material. It is a metallurgical copper produced by pyrometallurgical refining of raw metallurgical copper. Other elements are as follows: Bi (max. 0.0005%), Pb (max. 0.005%), O (max. 0.040%) and others 0.03%. The company Feropol, Ltd. (Bystřička, Czech Republic), supplier of copper metal products, supplied the semi-finished products for the production of samples with the required material composition, while the original dimensions of bars with a diameter of 10 mm and a length of 3000 mm were adjusted to partial samples used in experiments.

The method of perpendicular extrusion angle *φ* = 90° (ROUTE A) without sample rotation was chosen as the method used. The values of mechanical parameters in average values were:

1. Young’s modulus of elasticity *E_mat_* = 84,500 MPa;
2. Yield strength *Re* = 110 MPa;
3. Strength limit *Rm* = 216 MPa;
4. Failure limit *R_fr_* = 90 MPa;
5. Density *ρ* = 8653 kg∙m^−3^;
6. Input diameter of structural grain *D_gro_* = 12.74 µm.

Due to the maximum discharge force of the selected device *F_ec_*(*max*) = 1000 kN, a limited number of extrusions were realized, that is, *n* = 6. The measurement results were numerically processed and were also graphically illustrated using adequate graphs (see below).

In the presented experiment, route A (Figure 1) was used with an extrusion channel angle of 90° with a length of extrusion channel of 165 mm. The initial length of the samples *L_s_* was 100 mm. The samples were cleaned and lubricated with a Mo_2_S grease paste for each pass. The measurement was performed at a room temperature of 21 °C. The selected velocity through the channel was 1 mm∙s^−1^, at a discharge of up to *F_ecn_* = 1000 kN. The matrix has two intersecting channels: a vertical channel located in the direction of the mandrel axis and a horizontal channel perpendicular to the mandrel axis, where Φ = 90°. The intersection of the channel has a radius R = 5 mm on the outside and r = 0 mm on the inside. The matrix is non-removable. The die and mandrel are made of tool steel class 19, which is hardened and tempered to a hardness of 70 HRC.

Current knowledge confirms that although extrusion at higher temperatures is much easier, in order to obtain a high-quality fine-grained structure, the material must be extruded at the lowest possible temperature at which cracks do not yet form in the material. Keeping the extrusion temperature low ensures the potential to achieve a uniform structure with the smallest possible grain size and, at the same time, with the largest proportion of high-angle grain boundaries [10].

### 2.2. Measurement of Ultrasonic Speed

The Olympus Epoch 600 ultrasonic flaw detector (Olympus NDT, Waltham, MA, USA) was used to determine the speed in the samples. Measurement of the propagation velocity of the elastic waves was carried out through the measurement of the frequency of re-circulation of ultrasonic signals in the material. Sending and receiving of the ultrasonic waves in the material was performed by piezo-electric ultrasonic transducers of longitudinal waves (transducer XF1). The results of speed measurement at each point on the sample were recorded and stored in the memory of the device microprocessor. Ultrasonic velocity measurements were made continuously over 1 cm of sample length, and 10 measurements were taken after each extrusion.

### 2.3. New Method for Mechanical Analysis of Extrusion Functions

Based on the authors’ relatively extensive experience and evidence in the study of the concerned area [35,36,37,38], a new method of predicting the structural and mechanical change of materials during extrusion by the method of multiple plastic deformations was developed.

**Figure 2:**
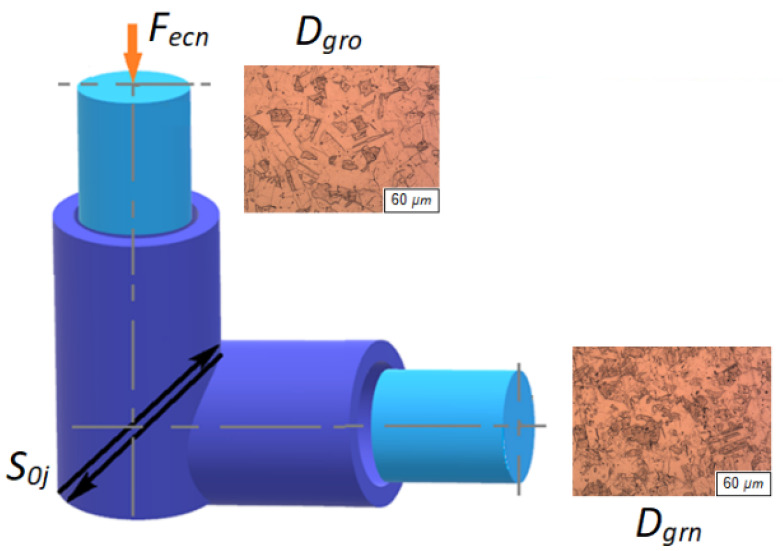
Principal scheme for the derivation of the equation.

The calculation is based on the authors’ equilibrium equation for the ECAP mechanism (1) and other equations expressed from this equation (Figure 2). By calculating according to Equation (1), the required number of extrusion cycles and the required amount of pressing force can be analytically determined in order to achieve the desired material reinforcement due to the reduction of the structural grain diameter of a particular material. In Equation (1), the stress is *σ_ecn_* (MPa), which is determined as the measured force *F_ecn_* (kN) acting on the specific surface *S* of the ECAP used. Equation (1) is used to check the objectivity and accuracy of measurements. Both members in the equation must be equal. The proposed material equilibrium equation expresses the compactness of the sample material (1):

(1) $$\frac{\sigma_{ecn}}{Re}-\frac{\varepsilon_{PLn}}{n}=0$$

where *Re* is yield strength (MPa), *ε_PLn_* is relative deformation of the structural grain (-), *n* is number of extrusions (-).

Figure 2 shows a diagram of the process on the basis of which the equilibrium equation was created. The arrows indicate the shear mechanism of the grain size change. Equation (1) is created from the relationship between the input physical and technological parameters. The compressive force generated by the device as the force *F_ecn_* acting on the material (with material characteristic *Re*) related to the cross-section of the extrusion channel as well as the ratio of the original and compressed grain areas present the cause and effect of deformation of the material structure in relation to the number of passes. The equilibrium equation can thus practically serve to dimension the technological conditions of the extrusion process. Based on the measurement of the values of the force *F_ecn_* and the yield strength *Re*, the number of extrusions can be predicted, that is, the change in the size of the original grain to the desired size can be predicted. Alternatively, we can also predict what force sizes we need to enter for the desired grain size; at the same time, we are limited by the real possibilities of the device (geometric dimensions of the channel, press power, etc.).

In the general concept, the equilibrium Equation (1) represents the volume deformation, where the ratio of the force *F_ecn_* to the area *S* represents the volume stress, *Re* the material coefficient and the ratio *ε_PL__n_*/*n* corresponds to the volume deformation.

The relative deformation can also be expressed by another fundamental equation, namely (2):

(2) $$\varepsilon_{PLn}=\frac{D_{gr0}}{D_{grn}},$$

where *D*_*gr*0_ is the grain size before extrusion (µm) and *D_grn_* the grain size after extrusion (µm). Then the required number of extrusions *n* (-) for a given material and a given ECAP design can be expressed by Equation (3):

(3) $$n=Re\cdot \frac{\varepsilon_{PLn}}{F_{ecn}}\cdot S_{0j}$$

where *S*_0*j*_ is a unit area of the sample (mm^2^).

According to (1), other important connections in the process can be expressed as (4):

(4) $$F_{ecn}=Re_{n}\cdot \frac{\varepsilon_{PLn}}{n}\cdot S_{0j}$$

where *Re_n_* is the yield strength after individual extrusions.

The parameter *D_grn_* according to (2) also corresponds to the equation from Hall–Petch Equation (5):

(5) $$D_{grn}={\left(\frac{k_{hp}}{\sigma_{hp}-Re}\right)}^{2}$$

where *k_hp_* is the Hall–Petch constant (MPa), *σ_hp_* is the Hall–Petch stress (MPa).

The newly derived equations were confronted with the procedures according to Hall–Petch [2] and according to the theory published in [39,40] to show the new possibilities and ways of using the theoretical principle of general relative deformation *ε_n_* from the generalized Hooke’s law. 

The increase in Hall–Petch tension [2] is given by Equation (6):

(6) $$\sigma_{hpn}=Re+\frac{k_{hp}}{\sqrt{D_{grn}}}$$

The mechanical power of the ECAP *N_ecn_* (kW) can be evaluated for individual extrusions by Equation (7):

(7) $$N_{ecn}=F_{ecn}\cdot v_{pecn}$$

where the optimal rate for experimental material *v_pecn_* (m∙s^−1^) is given by (8):

(8) $$v_{pecn}=10^{3}\cdot \frac{\sqrt{10^{-3}\cdot D_{gr0}}}{60\cdot \sqrt{E_{matn}}}$$

where *E_matn_* is the altered modulus of elasticity (MPa). 

## 3. Results and Discussion

### 3.1. Measured Parameters

**Figure 3:**
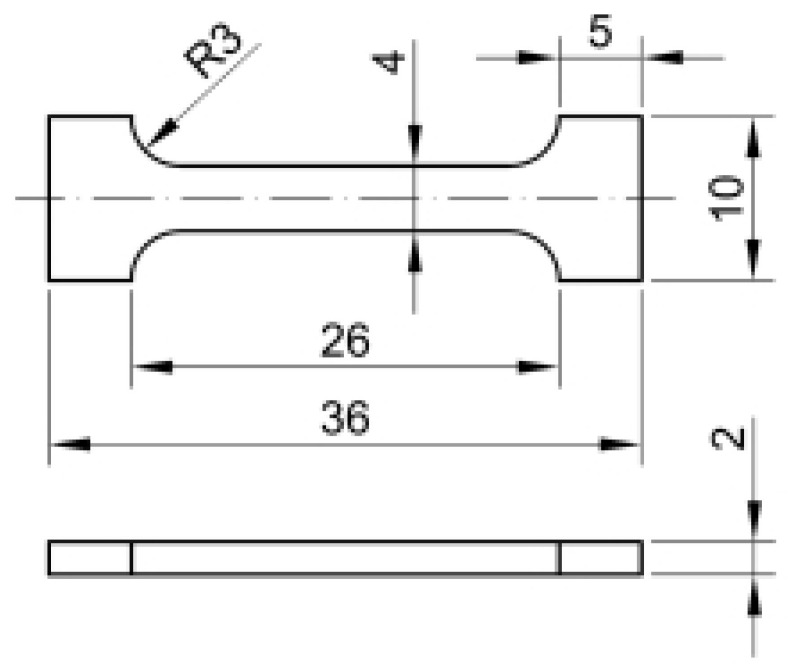
Drawing of the test bar used in the tensile tests.

**Figure 4:**
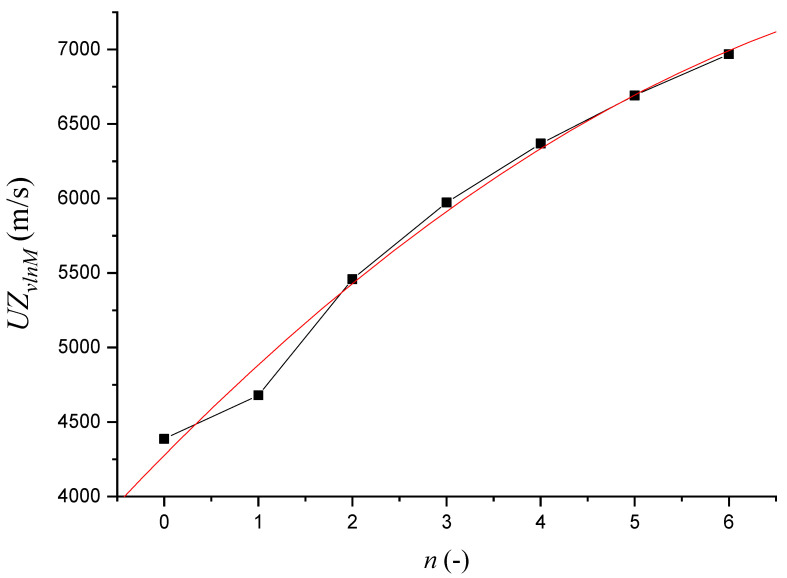
Dependence of ultrasonic speed *UZ_vlnM_* on the number of extrusions *n*.

**Figure 5:**
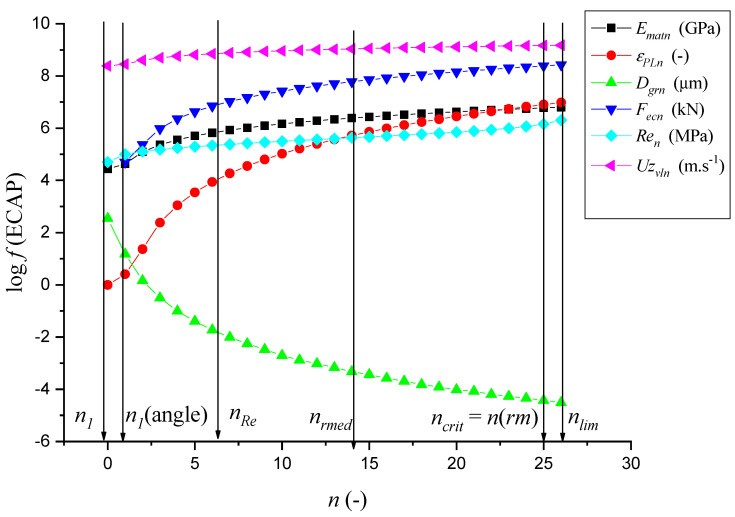
Results from Table 1 and Table 2 in logarithmic measure.

**Figure 6:**
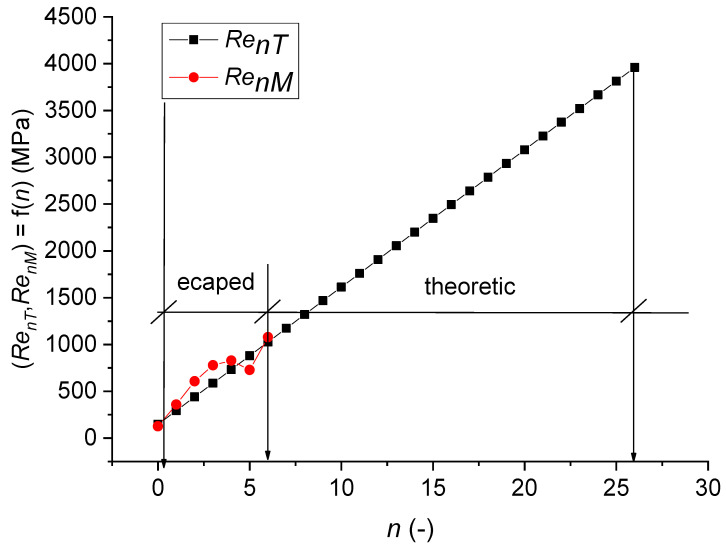
Dependence of (*Re_nT_*, *Re_nM_*) = f(*n*).

**Figure 7:**
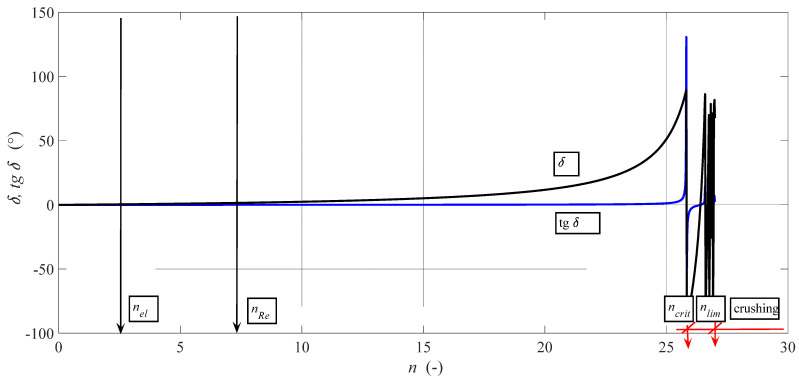
Angle of internal friction.

**Figure 8:**
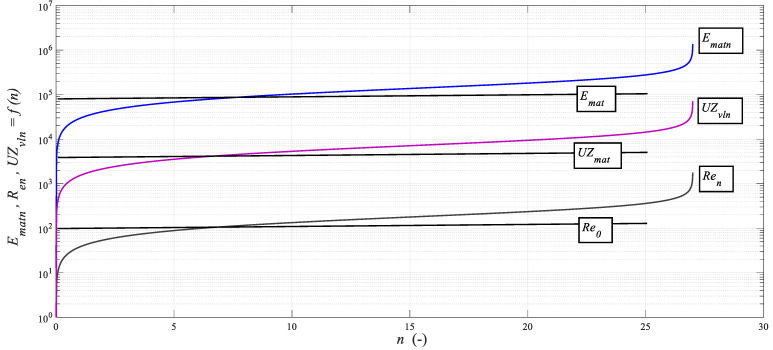
Dependence of functions *E_matn_*, *Re_n_*, *UZ_vln_* = f(*n*).

The experimental work results (Table 1) and prediction parameters (Table 2) presented in the figures (Figure 3, Figure 4, Figure 5, Figure 6, Figure 7 and Figure 8) represent comparisons of the measured and theoretically predicted values. The change of process functions can be seen from the course of individual functions after up to 26 extrusions. The number of extrusions was taken from the calculation according to Equation (3) based on the intention to achieve a nano-dimension in grain diameter *D_grn_* = 10 nm, namely for ECAP-A and material parameters: Cu 99.9%, *D*_*gr*0_ = 12.74 µm, *E_matn_* = 84,500 MPa, ultrasound velocity on the neutral plane *UZ_vlo_* = 4387 m∙s^−1^. The *UZ_vln_* symbol is then the change in speed according to the number of extrusion cycles *n*. In Table 1, a capital M means measured parameters.

The samples were extruded through a hydraulic press through a 90° angled channel for individual extrusions 1 to 6 (Table 1). A total of 30 samples were generated in this way, with the ultrasound velocity *UZ_vlnM_* (m∙s^−1^) first being measured on an Olympus Epoch 600 instrument as described in subchapter 2.2. In order to measure the forces *F_ecnM_* (kN) in the ECAP process, a resistance strain gauge sensor type 3/350 XK 11 designed at VŠB-TU Ostrava was used, which measures with a range of up to 1000 kN and with an accuracy of 0.5%. Its rated resistance is R1–R8 = 350 Ω.

An Olympus GX51 inverted metallographic microscope was used for visual observation of metallographic grinding and polishing. Thus, the grain sizes *D_grnM_* were obtained, and then the parameter *ε_PLn_* was evaluated according to Equation (2) and the parameter *ε_PLn_* was measured (Table 1).

Tensile tests of samples were (Figure 3) performed in a certified testing laboratory of VÚHŽ a.s., Dobrá, while for each evaluated group of samples, five pieces of test specimens were produced after individual extrusions. Young’s modulus of elasticity *E_matn_* was determined in accordance with ASTM E-111. The determination of the yield strength *Re_M_* was performed methodologically by modifying the standard EN 10002-1. The deformation rate used was in accordance with the standard 0.0025 $s^{-1}$. Due to the size of the sample, no extensometer was used (deformation sensing was performed by electronic sensing of the crossbar at a magnification of 250× and with an accuracy of ±0.5 µm). All calculations of mechanical values were also performed in accordance with EN 10002-1. Table 1 shows the averaged values for the individual parameters.

Table 1 plots the dependence between *UZ_vlnM_* (m∙s^−1^) on *n* (-) (Figure 4) and presents a polynomial Equation (9) with the total accuracy of measurement R^2^ = 0.99.

(9) $${UZ}_{vlnM}=4419.883+538.063\cdot n-23.380\cdot n^{2}+0.403\cdot n^{3}$$

### 3.2. Prediction Parameters

Based on Equation (10), the *UZ_vlnP_* (m∙s^−1^) values in Table 2 were predicted. In Table 2, capital P means the predicted parameters. These parameters are calculated according to the equations for *ε_PLnP_* (2), *D_grnP_* (5), *F_ecnP_* (4), after expressing *Re* from Equation (1), *Re_P_* and *U_zvlnP_* (9) were calculated.

For further analyses, the individual dependencies (Figure 5) from Table 1 and Table 2 were plotted to make it possible to monitor and interpret individual relationships and interactions between parameters.

For clarity, the individual courses in Figure 5 were converted to a logarithmic measure, so it was clear to see that the yield strength changes with the individual extrusions and thus gradually increases. Furthermore, with the generated value of *UZ_vln_*, the absolute size of the compressed grain, that is, the relative size of the compressed grain to the initial grain size, decreases significantly, simultaneously with the increase of the applied compressive force of the extruder.

In Figure 5, the following applies to the individual levels, the positions of these levels being given by the conversion of the deformation length to the number of extrusion cycles *n* = *h_def_*∙*k_hn_*, where *h_def_* is the deformation length, *k_hn_* is the conversion from the deformation length to the number of extrusions *n*, or *n_lim_* = *K_plmat_*∙*k_hn_*, or *n_o_* = *h_o_*∙*k_hn_*. For Cu 99.9, *k_hn_* = 0.193 (-), *K_plmat_* = 139.72 mm, *h_o_*= 37.76 mm, *n_o_* = 7.29 (-).

In Figure 5, lines are marked by verticals for important limits in the number of extrusions *n* and in the mechanism of extrusion of the sample through the channel. They are given to the nearest tenth to express the sample stress-strain degree in the channel as closely as possible. Thus, we can control the sample material’s instantaneous mechanical state more accurately according to the diagram for *σ*-*n*, which is shown above in Figure 5. The presented solution is of user importance for dimensioning structures, for checking the level of grain size refinement and mechanical parameters. The individual sections in Figure 5 are:
*n*_1_Represents a high resistance of the surface layer.*n* (angle)The level of the beginning of the effect of the channel break (represents the influence of the ECAP technology knee).*n_Re_*Level of exceeding the yield strength *Re* of the material.*n* (rmed)Level of mean number of extrusions n(lim)/2.*n_cri_*_t_ = *n* (rm)Level of exceeding the yield strength *Rm* of the material (delimited zone of structural deformations).*n* (lim)Level of reaching the limit deformation capacity (zone of decomposition of structural bonds).

Figure 6 presents the dependence of the yield strength of the theoretically predicted *Re_nT_* and the measured *Re_nM_*, depending on the number of extrusions *n*.

The inputs to the algorithm for MATLAB are: Young’s modulus of elasticity *E_mat_* in MPa, or possibly ultrasonic speed *UZ_vl_* (m∙s^−1^) or yield strength *Re* (MPa), the required number of extrusion cycles *n* (-), input size of the structural grain *D*_*gr*0_ (μm) and the diameter of the extruded sample *D*_0_ (mm). The equations entering the calculation are the same as in the algorithm for Excel, so the resulting data are the same as well. This expresses the agreement of the distribution of the main functions according to the extrusion *n* = 26.

The necessary equations for the calculation by the MATLAB program were algorithmically arranged so that they follow each other adequately as a result. The algorithm produces all the required data at once and in a very short time. Data volume requirements can be added operatively and interactively. The resulting data can be produced both in tabular numerical form and in graphs.

The resulting values according to the Excel and MATLAB algorithms are identical because, in both cases, the same composition of equations were used.

### 3.3. Analytic Approach to the Solution

Analytically, we were forced to assume the homogeneity of the material. The influence of genetic inhomogeneity is revealed only by loading in the laboratory and, unfortunately, most often only in operational practice with destructive consequences. In our theoretical and application works, we have traditionally introduced limiting factors, for example, in the parameters of the critical length of mutual deformation displacement of structural layers *Y_ret_* (mm), it can also be the deformation displacement of planes in the crystal lattice under loading. Another critical factor in our works is the angle of internal friction *δ* (°) and its trigonometric derivatives cos *δ* or tg *δ* (Figure 7). These factors are best studied in sections with flexible tools such as abrasive waterjet (AWJ), laser or ultrasound [41,42]. Qualitative differences are easily measurable on sections with these tools, often visible to the naked eye; it is easy to detect the so-called neutral plane, often quoted in our works, or the depth of cut *h*_0_ (mm). Analogies of *h*_0_ for a fixed tool are the neutral depth of cut *a_po_* (mm) and, for example, for ECAP, it is the neutral number of extrusions *n*_0_ (-). These parameters accurately locate the position, the level of reaching the yield strength *Re* (MPa) and the important parameters at the yield strength *Re* for each material. This is both the modulus stress and strain stress level, where we often deal with the relative deformation *ε*_0_ (-), or the grain diameter *D*_*gr*0_, or roughness of *Ra*_0_ [37]. These parameters are evaluated at the neutral plane. The limit parameters for the graphical dependence of individual parameters on *n* document the achievable extrusion of *n_lim_* for a specific material. This is demonstrably given by achieving the so-called limit deformation capacity of the material given in terms of structural grain size by the plasticity constant *K_plmat_* = *D_grn_*∙*n*/*Y_ret_* (mm). However, with the ECAP technology, we reached the level of the critical number of extrusions *n_crit_*, where the critical angle of internal friction is reached by the value arc *δ* = 90°. These values are of great analytical significance to us and to theory and practice in general.

For other materials, the conversion according to *k_ec_* = *K_plmat_*/*K_plmatREF_* can be demonstrably used, where *K_plmatREF_* is the reference value, and the value *K_plmatCu_* = 139.72 mm can be calculated as the reference gateway. The computational algorithm represents Equilibrium Equation (1), where the main functions of interest are defined. Additional functions can be defined according to the scheme of values and functions to *n_o_*, which is equal to the measured or tabular value. The deformation of each of the additional functions, whether increasing or decreasing in ECAP, is given by the alteration by multiple of *ε_PLn_*_._ For example, alteration *E_matn_* = *E_mat_*∙*ε_PLn_*(*E_mat_*) or *UZ_vln_* = *UZ_mat_*∙*ε_PLn_* (m∙s^−1^) according to Figure 8. Where appropriate, the courses of the functions were converted to a logarithmic measure.

The complexity of the topic, compared to the fundamental simplicity of the measurement itself, brings a number of interdependent variables. This can best be expressed by an implicit equation related to the significance of the structural grain size *D_gr_* (µm). In the form of a common relationship to the speed of the ultrasound wave, the implicit equation will be given below in answer to a question on the use of ultrasound.

### 3.4. Data Distribution Calibration Graphs

**Figure 9:**
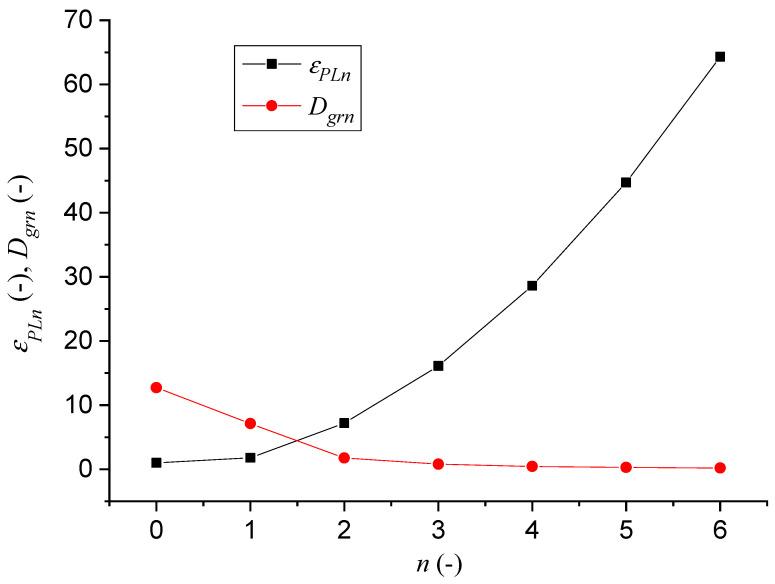
Calibration graphs of dependence (*ε_PLn_*, *D_grn_*) = f(*n*) for *n* = 0, 1, 2, 3, 4, 5, 6.

Figure 9 shows the dependence of the relative elongation *ε_PLn_* and the grain size *D_grn_* as a function of the number of extrusions *n*. There is a visible intersection, which corresponds to the level characterizing the situation occurring in a “rectangular bend”, or in general, in “a bend at an angle”.

## 4. Comparison and Verification of Results

Experimental and analytical procedures were validated according to the authors’ basic theoretical assumptions on the integrity of mechanical, stress, deformation and structural parameters of materials. The size of the structural grain is one of the most important structural parameters of materials. The obtained results confirm the assumptions and provide suitable material for professional discussion. In the authors’ opinion, the important benefits for theory and practice include, in particular:

- Derivation of the equilibrium equation for extrusion by the ECAP method (1);
- Derivation of new Equations (1) and (7)–(18) needed for solving the problem by the ECAP method;
- Algorithmizing of calculations for Excel and Matlab programs.

### 4.1. Comparison and Verification of the Results According to the Velocity of Longitudinal Ultrasound Waves

The velocity of the longitudinal ultrasound waves *UZ_vln_* is given in more detail in correlation with the functions (Table 1 and Table 2). The number of extrusions was taken from the calculation according to Equation (10), based on the intention to achieve a nanoscale at a grain diameter *D_grn_* = 10 nm. As can be seen from the newly obtained correlation Equations (10)–(17), the functions related to the velocity of the longitudinal ultrasonic wave *UZ_vln_* (m∙s^−1^) can be fully utilized in the calculations for the ECAP technology.

**Figure 10:**
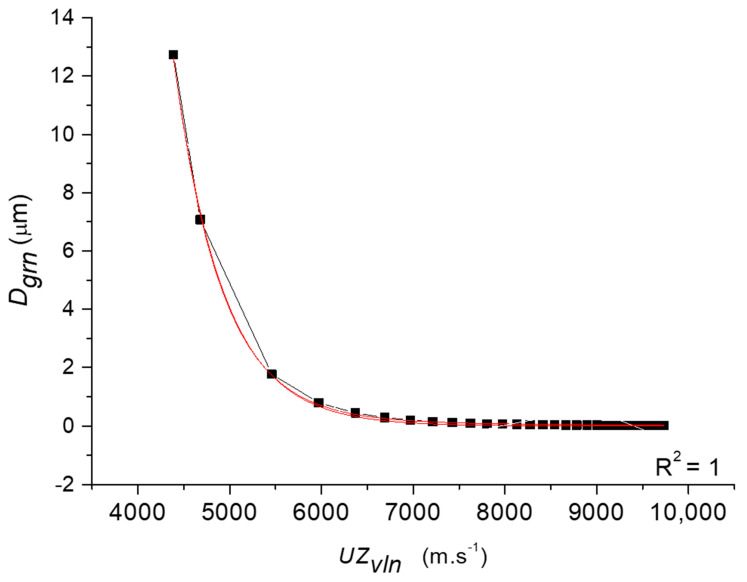
Correlation relation *D_grn_* = f(*UZ_vln_*), which is given by Equation (11).

Figure 10 shows the logarithmic dependence of the parameters of grain size *D_grn_* (10), power *N_ecn_* (11), number of extrusions *n* (3) and the relative deformation of grain size *ε_PLn_* (2) on the longitudinal velocity of ultrasonic waves *UZ_vln_* (m∙s^−1^).

For the dependence of the grain size *D_grn_* (µm) on the longitudinal velocity of ultrasonic waves *UZ_vln_*, Equation (10) applies:

(10) $$D_{grn}=43,406.546\cdot e^{\left(\frac{-\;UZ_{vln}}{541.680}\right)\;}-852.994\cdot e^{\left(\frac{-\;UZ_{vln}}{602.145}\right)}$$

Figure 10 shows the dependence of the grain size *D_grn_* on the velocity of the longitudinal ultrasonic waves *UZ_vln_*.

The power *N_ecn_* (kW) dependent on the longitudinal velocity of the ultrasonic waves *UZ_vln_* is given by (11):

(11) $$N_{ecn}=0.083+5.669\cdot 10^{-5}\cdot UZ_{vln}-1.443\cdot 10^{-8}\cdot UZ_{vln}^{2}+6.457\cdot 10^{-12}\cdot UZ_{vln}^{3}$$

The number of extrusions *n* (-) depending on the longitudinal velocity of the ultrasonic waves *UZ_vln_* is presented by Equation (12):

(12) $$n=-20.911+0.011\cdot UZ_{vln}-1.966\cdot 10^{-6}\cdot UZ_{vln}^{2}+1.406\cdot 10^{-10}\cdot UZ_{vln}^{3}$$

The relative deformation *ε_PLn_* (-) depending on the longitudinal velocity of the ultrasonic waves *UZ_vln_* is given by (13):

(13) $$\varepsilon_{PLn}=0.110\cdot e^{\left(-\;\frac{UZ_{vln}}{1035.076}\right)}-\;20.928\cdot e^{\left(-\;\frac{UZ_{vln}}{1.186}\right)}$$

**Figure 11:**
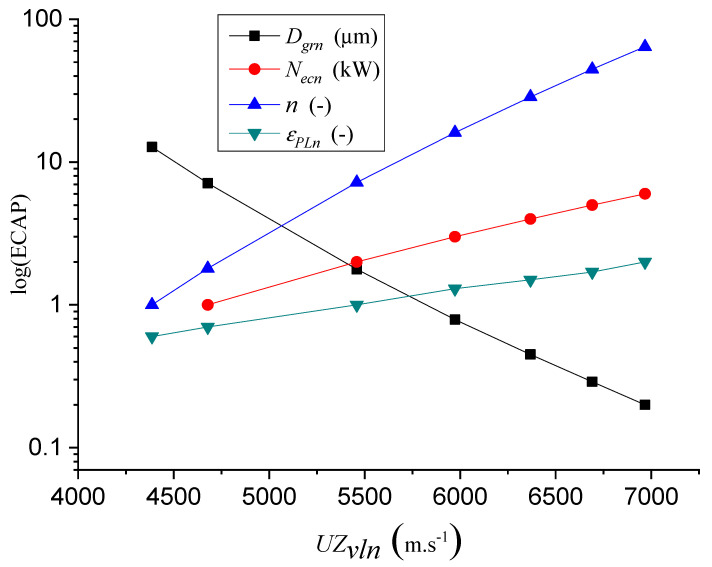
Correlation relations log f(ECAP) = f(*UZ_vln_*).

Figure 11 shows the dependence of the grain size *D_grn_*, *N_ecn_* (7), *n*, *ε_PLn_* on the velocity of the longitudinal ultrasonic waves *UZ_vln_*.

**Figure 12:**
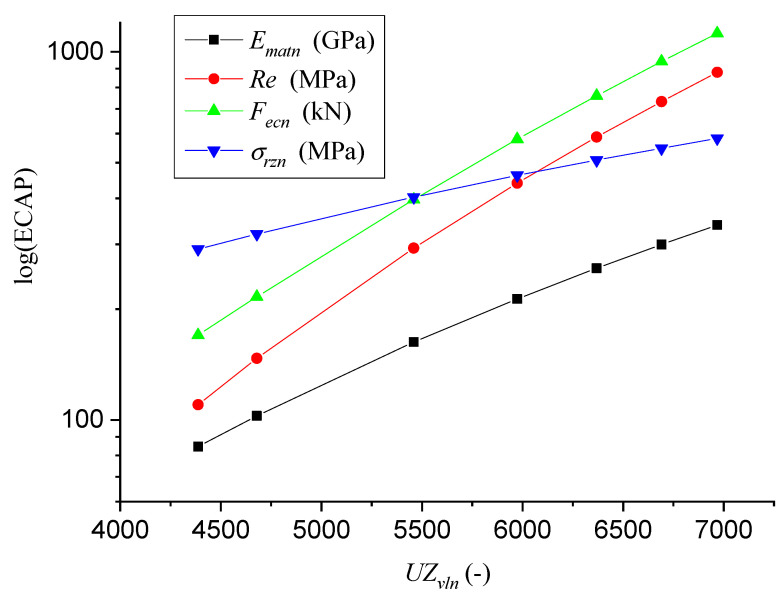
Correlation relations LOG f(ECAP) = LOG f(*UZ_vln_*).

Figure 12 presents the logarithmic dependences of Young’s modulus of elasticity *E_matn_* (14), the yield strength *Re_n_* (15), the force *F_ecn_* (16) and the actual stress *σ_rzn_* (17).

For the dependence of Young’s modulus of elasticity *E_matn_* on the longitudinal velocity of the ultrasound *UZ_vln_*, Equation (15) applies:

(14) $$E_{matn}=1.431-5.23363\cdot 10^{-4}\cdot UZ_{vln}+6.234\cdot 10^{-8}\cdot UZ_{vln}^{2}+9.976\cdot 10^{-10}\cdot UZ_{vln}^{3}$$

The yield strength *Re_n_* depending on the longitudinal velocity of the ultrasound *UZ_vln_* is presented by Equation (15):

(15) $$E_{matn}=1.431-5.23363\cdot 10^{-4}\cdot UZ_{vln}+6.234\cdot 10^{-8}\cdot UZ_{vln}^{2}+9.976\cdot 10^{-10}\cdot UZ_{vln}^{3}$$

The force *F_ecn_* (kN) depending on the longitudinal velocity of the ultrasound *UZ_vln_* is given by (16):

(16) $$F_{ecn}=-2324.342+1.369\cdot UZ_{vln}-2.804\cdot 10^{-4}\cdot UZ_{vln}^{2}+2.222\cdot 10^{-8}\cdot UZ_{vln}^{3}$$

The actual stress *σ_rzn_* (17) depending on the longitudinal velocity of the ultrasound *UZ_vln_* can be calculated according to Equation (18):

(17) $$\sigma_{rzn}=10^{-3}\cdot \frac{D_{grn}}{D_{gr0}}\cdot \frac{1}{E_{matn}}$$

where *σ_rzn_* is the stress that causes the structural grain to deform (MPa).

(18) $$UZ_{vln}=4419.883+538.063\cdot n-23.380\cdot n^{2}+0.403\cdot n^{3}$$

### 4.2. Control and Verification Comparison Results by Ultrasound and by Equilibrium Equation

The results by ultrasound equation and by equilibrium equation were plotted together in one graph. Both independent calculation methods evidently show identical results. Figure 10 depicts a partial correlation relationship *D_grn_* = f(*UZ_vln_*), which is given by Equation (10) and shows, in an exemplary manner, regression tightness R^2^ = 1. The same is true after plotting other parameters represented in the material Equation (3) in their relationship to the longitudinal velocity of ultrasonic waves *UZ_vln_*. This illustrates the following Figure 10 showing the logarithmic dependence of the parameters of power *Fecn* (16), number of extrusions *n* (12) and the relative deformation of grain size *ε_PLn_* (2) on the longitudinal velocity of ultrasonic waves *UZ_vln_*, calculated, for example, by (18) as a dependence of *UZ_vl_* = *f*(*n*).

### 4.3. Comparison of Author’s Own Investigations with Literature

**Figure 13:**
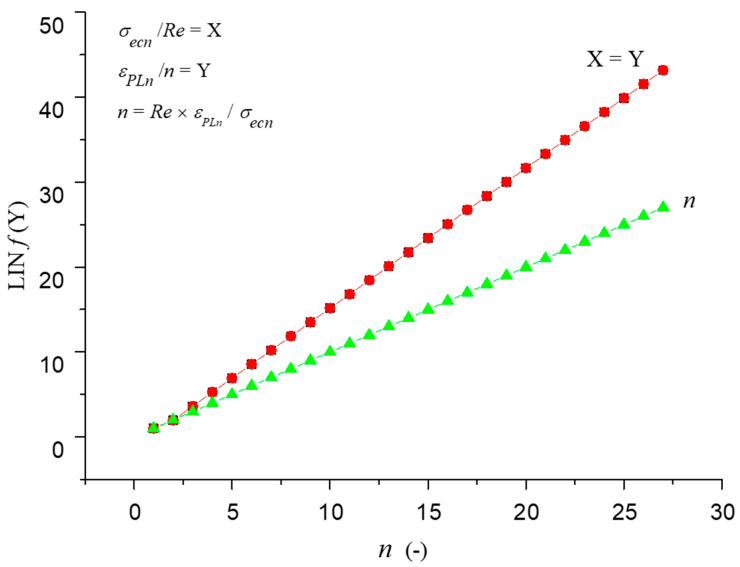
Check of the equilibrium Equation (1).

**Figure 14:**
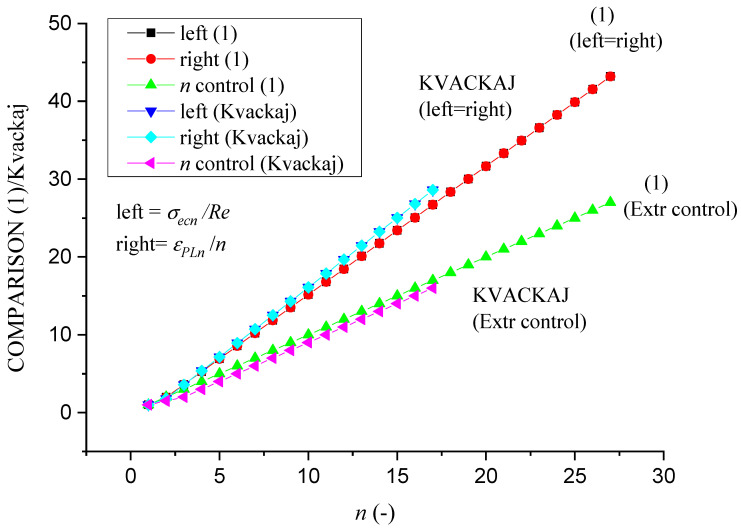
Comparison of data from our equilibrium equation with measured data by Kvačkaj.

**Figure 15:**
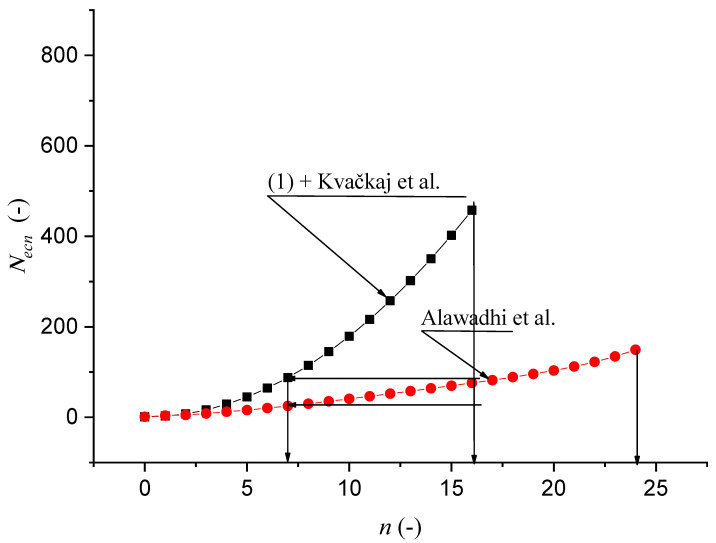
Comparison of the performances of equilibrium equation.

Figure 13 represents the check of the equilibrium Equation (1). Comparison of data from our equilibrium equation with measured data by Kvačkaj is shown in Figure 14 and with Kvačkaj, Mashal in Figure 15.

The comparison of the left and right sides of the equation is declared in Figure 14. It is evident that for the left side, there is a smaller difference between the data up to 5% between the equilibrium Equation (1) and Kvačkaj. On the right side, there is a relative identity between these data.

Figure 15 represents a comparison of the performances of equilibrium Equation (1), Kvačkaj et al. (16 extrusions) [43], which are identical, and Alawadhi et al. (24 extrusions) [44], where the difference occurs, due to, for example, the use of a different force, material: oxygen-free copper) with a different material structure and chemical composition.

For these conclusions, we proceed, for example, from the newly derived equation of equilibrium (1) according to the increment or loss of grain diameter and function values in relation to the number of extrusion *n*.

**Figure 16:**
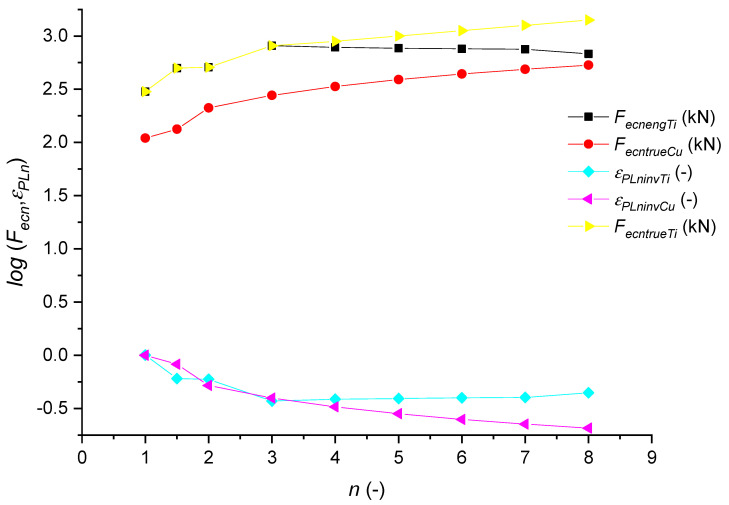
Comparison of force and relative deformation for materials Cu/Ti.

We checked this assumption according to an experiment on titanium Ti (*E_ma_*_t_ = 162,800 MPa; *K_plmat_* =10^12^/*E_mat_^2^* = 37.65 mm). The ratio of material constants of titanium and reference copper is given as follows: *IND_kpl_* = *K_plmatREF_*/*K_plmatTi_* = 139.72/37.65 = 3.71. This ratio remains constant. This was proven analytically and experimentally. Figure 16 is plotted according to titanium data from the literature [45] and Cu data from the present paper. Greger et al. measured the engineering values of the stress and these were converted to true values for the force over the deviation angle cos *δ* according to the patent [37]. The ratio of the Ti/Cu curves represents a function of the *K_plmat_* ratio of both Cu/Ti materials. In Figure 15, *F_ecnengTi_* is the engineering force for Ti, *F_ecntrueTi_* is the true force for Ti, *F_ecntrueCu_* is the true force for Cu, *ε_PLnTi_* is the relative deformation for Ti (2) and *ε_PLnCu_* is the relative deformation for Cu (2).

## 5. Evaluation of Results and Discussion

The main result of the presented research can be considered the derivation of a new equation of material equilibrium (3) intended to strengthen materials with an ECAP type extrusion set. The proposed calculation can theoretically predict the required number of extrusion cycles and the required amount of pressing force exerted to reduce the selected material’s structural grain diameter. Thus, the desired strengthening of the selected material can be achieved without the material becoming inhomogeneous.

So far, the decision on the procedure by the extrusion method has been made only based on the operator’s subjective experience. The exact design of the extrusion works will objectively and practically make it possible to streamline the refinement of the selected material structure without the ECAP die being at risk of destruction.

In short, the benefits of the presented publication can be seen on three levels. From the theoretical point of view, it is the introduction, interpretation and verification of a new equation of equilibrium of the material subjected to compressive and shear force; the prediction of the grain size up to the limit fine-grained structure; and also the technological prediction of the maximum possible compressive force to be expended during the ECAP extrusion process, including the lack of more detailed analytical processing of the process mechanism.

The presented research deals in a relatively detailed and comparative way with the use of ultrasound. In this context, a very close correlation between the process functions of extrusion (1) and the speed of longitudinal ultrasonic waves was confirmed. The correlations in question were also processed into Equations (9)–(18). The practical significance of the proposed application of ultrasound lies in its operability, speed, cheapness and sufficient, easily achievable accuracy. The relationships of ultrasound to other important material parameters can also be used in other applications necessary for the dimensioning/design of load-bearing elements of buildings and machine structures, especially in terms of the suitability of the selection and the need for further refining of the material.

In connection with the advantages of using ultrasound for the ECAP method, it should be noted that technically, it is possible to provide continuous signal recording, signal transfer to a computer and immediate calculation of all process functions according to the equations presented here, thus acquiring a comprehensive report on the distribution of all monitored material parameters online.

## 6. Conclusions

The presented publication aims to contribute to the solution of problems in the field of materials engineering, namely by analytical description, and thus to verify the specifics of the behavior of technical materials depending on the change in the size of the structural grain towards nano-areas. The paper described the ECAP extrusion process with vertical extrusion without sample rotation in a comprehensive way, which is mainly a new method of the physical-mathematical modeling of stress-strain states of materials, including verification using ultrasound, due to cheap and express process diagnostics. The newly designed method allows predicting changes in the mechanical parameters of nanomaterials, with nano-level structural grain size. The main goal of this paper is to find new ways of utilizing the unique properties of nanoparticles and nanostructures in technical practice for the development of new materials necessary for the construction of technical equipment and systems.

The presented publication summarizes the primary innovative benefits of the solution, both for theory and for the laboratory and technical practice. In particular, it is the case of the derivation of the equilibrium equation for ECAP (1) and the description of new Equations (1) and (7)–(18). The new equations are mainly used to predict and understand the process in the refining of materials.

Verification based on static and mostly continuous measurement of the extruded sample using ultrasound provides the possibility of online digitization of measured data and laboratory reports.

## Figures and Tables

**Table 1:** Selected measured parameters for *n* = 〈0–6〉, where *E_matn_* is the altered *E_mat_*.

*n*	*E_matn_*	*ε_PLnM_*	*D_grnM_*	*F_ecnM_*	*Re_M_*	*UZ_vlnM_*
(-)	(GPa)	(-)	(µm)	(kN)	(MPa)	(m∙s^−1^)
0	84.5	1.00	12.740	0	110.00	4387
1	102.5	1.50	3.244	110	148.00	4679
2	162.7	3.93	1.177	216	164.30	5459
3	213.1	10.83	0.605	397	177.20	5973
4	258.2	21.05	0.367	579	188.40	6368
5	299.6	34.55	0.248	760	198.60	6691
6	338.3	51.38	0.178	942	208.20	6968

**Table 2:** Selected predicted parameters for *n* = 〈7–26〉.

*n*	*E_matn_*	*ε_PLnP_*	*D_grnP_*	*F_ecnP_*	*Re_P_*	*UZ_vlnP_*
(-)	(GPa)	(-)	(µm)	(kN)	(GPa)	(m∙s^−1^)
7	374.9	71.46	0.134	1123	217.40	7211
8	409.9	94.91	0.105	1305	226.30	7428
9	443.3	121.58	0.084	1486	235.00	7625
10	475.6	151.64	0.067	1668	243.70	7806
11	506.8	184.90	0.056	1849	252.40	7973
12	537.1	221.45	0.049	2030	261.10	8128
13	566.5	261.42	0.042	2212	270.00	8274
14	595.2	304.56	0.036	2393	279.20	8412
15	623.2	351.14	0.032	2575	288.60	8542
16	650.6	400.87	0.028	2756	298.60	8665
17	677.4	454.05	0.025	2938	309.00	8783
18	703.8	510.38	0.022	3119	320.30	8895
19	729.6	570.17	0.020	3301	332.50	9002
20	755.0	633.09	0.018	3482	346.00	9106
21	779.9	699.49	0.017	3664	361.40	9205
22	804.5	769.00	0.015	3845	379.30	9301
23	828.7	841.80	0.014	4026	401.10	9393
24	852.5	918.11	0.013	4208	429.60	9482
25	876.1	997.50	0.012	4389	470.90	9568
26	899.3	1080.42	0.011	4571	547.70	9652

## Data Availability

The data presented in this study are available on request from the corresponding author.
